# Supplementary material for: Circulating microRNAs for Early Diagnosis of Ovarian Cancer: A Systematic Review and Meta-Analysis
Source: Biomolecules. 2023 May 22;13(5):871. doi: 10.3390/biom13050871 (PMC10216356; doi:10.3390/biom13050871)

Supplemental Figure S1: Funnel plots

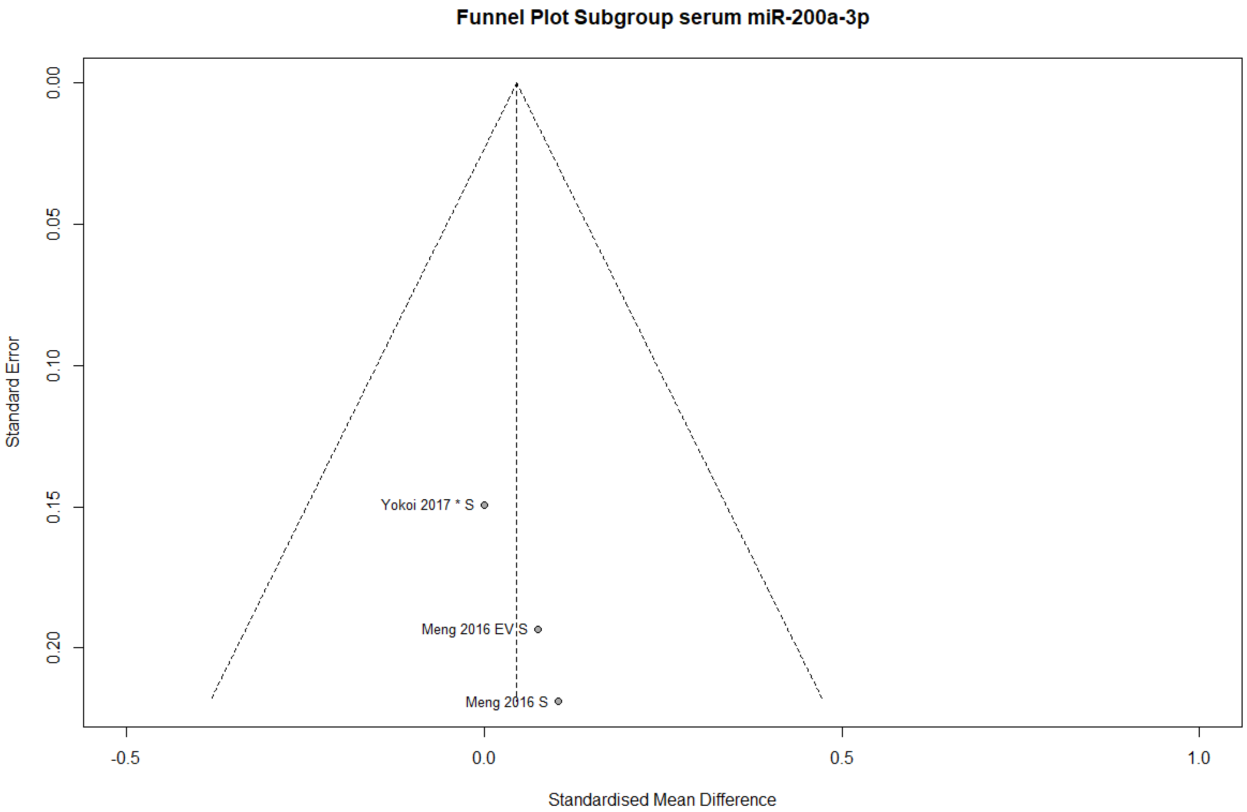

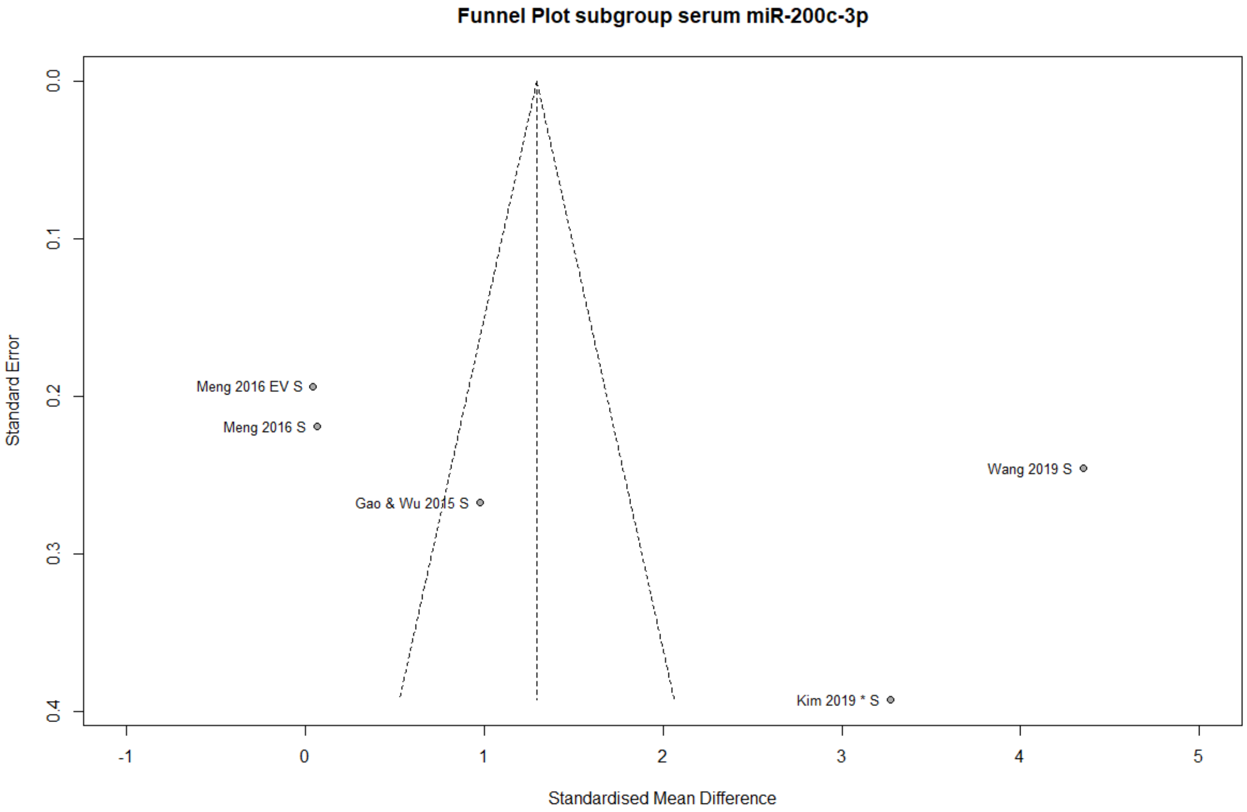

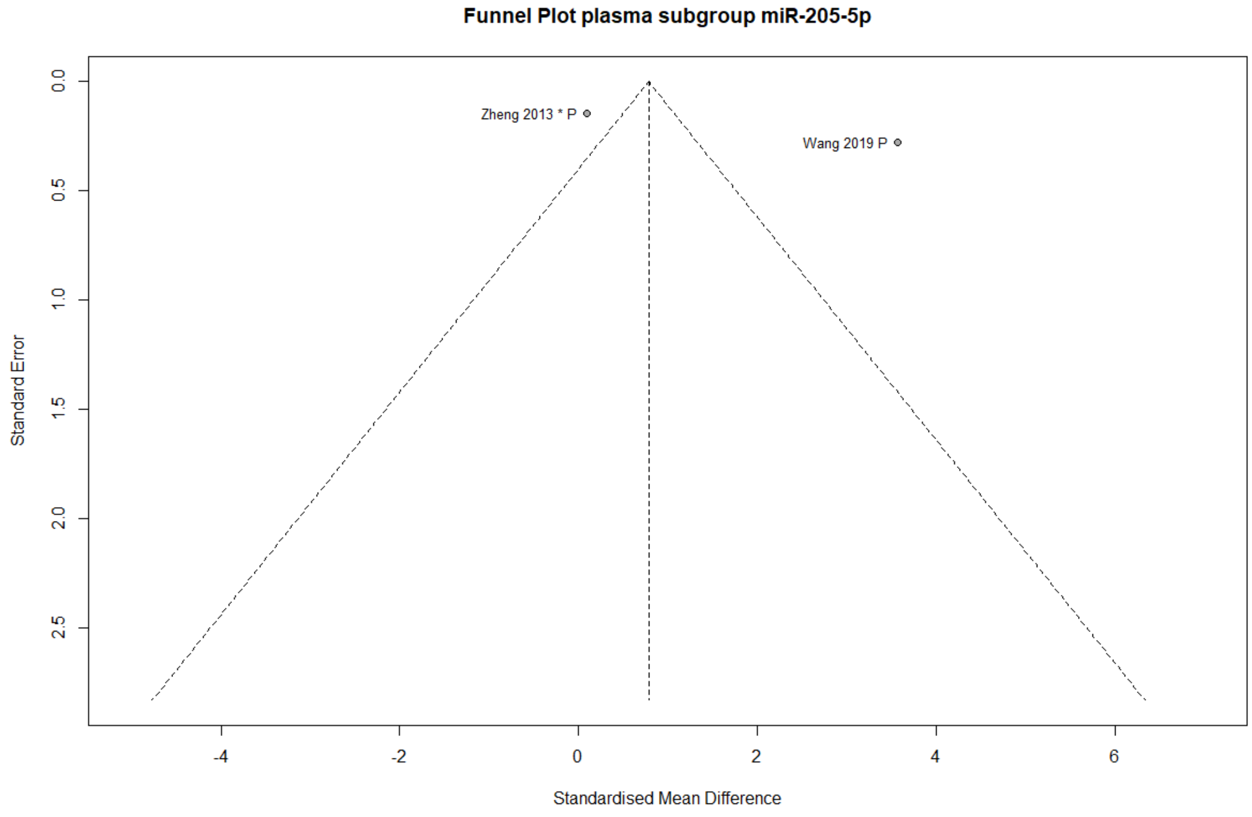
Funnel Plot plasma subgroup miR-205-5p
Zheng 2013 * P
Wang 2019 P
Standard Error
0.0
0.5
1.0
1.5
2.0
2.5
-4
-2
0
2
4
6
Standardised Mean Difference

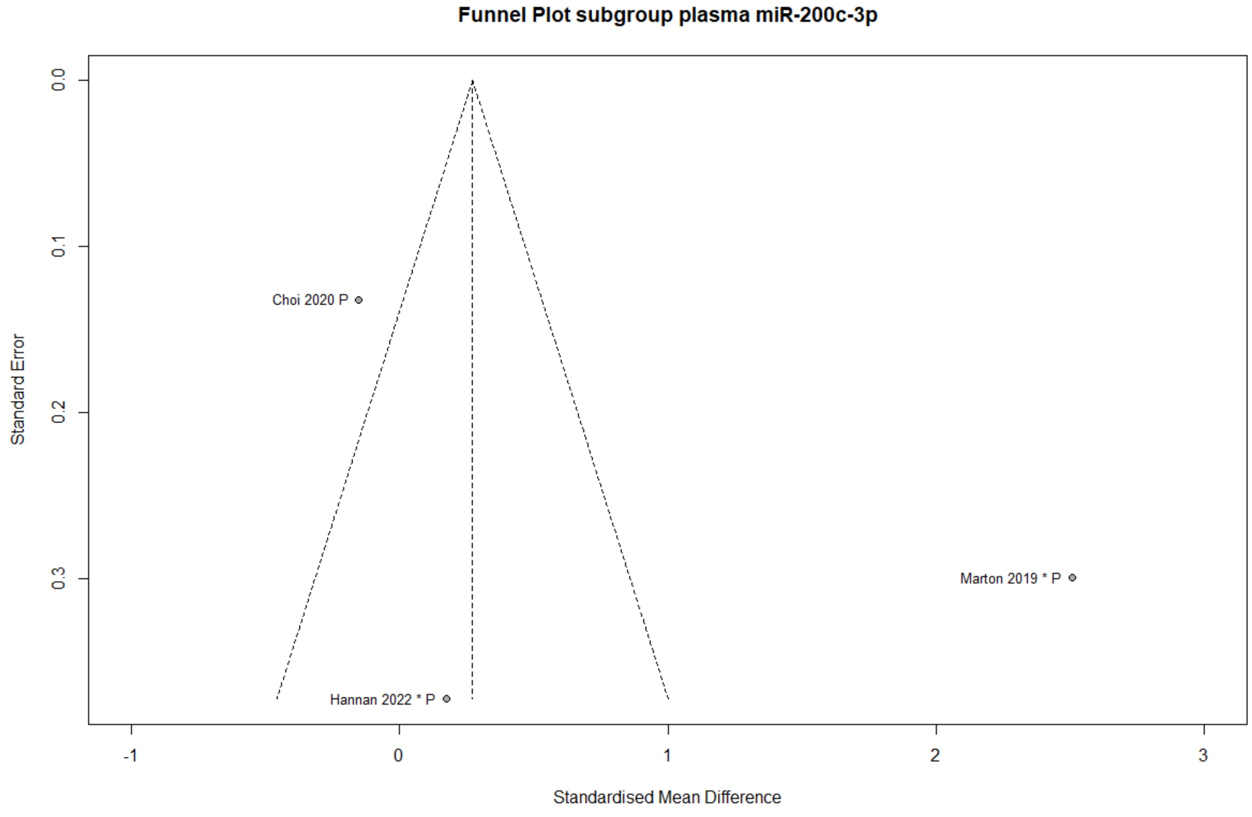
Funnel Plot subgroup plasma miR-200c-3p
Choi 2020 P
Marton 2019 * P
Hannan 2022 * P
Standard Error
0.0
0.1
0.2
0.3
-1
0
1
2
3
Standardised Mean Difference

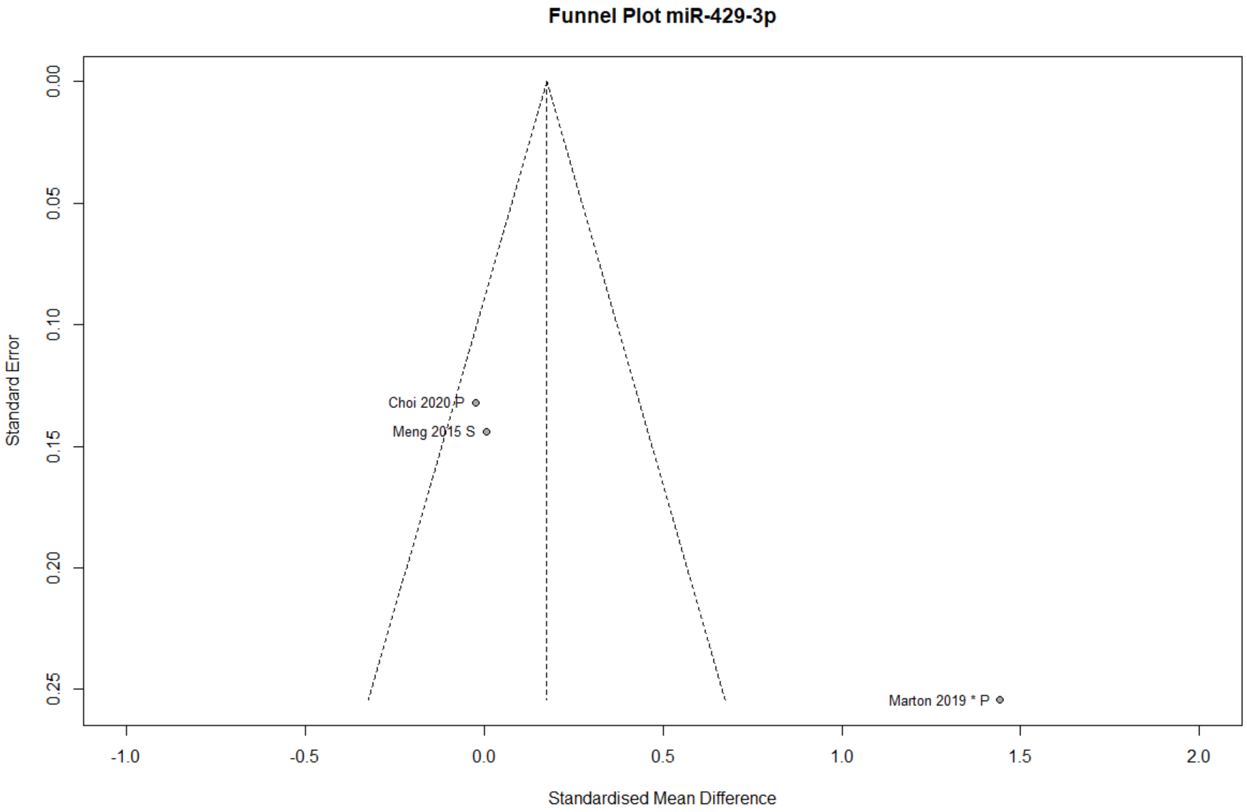
Funnel Plot miR-429-3p
Standard Error
0.00
0.05
0.10
0.15
0.20
0.25
Choi 2020 P
Meng 2015 S
Marton 2019 * P
-1.0
-0.5
0.0
0.5
1.0
1.5
2.0
Standardised Mean Difference

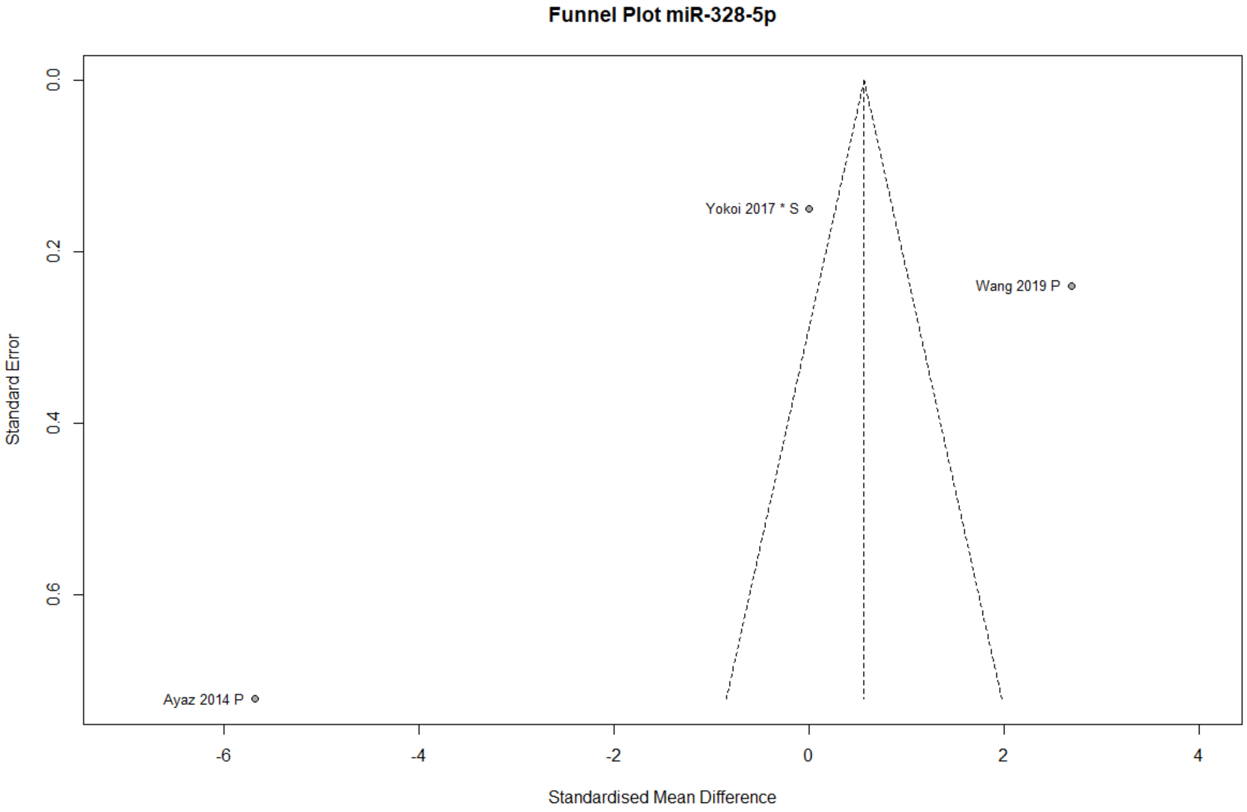
Funnel Plot miR-328-5p
Standard Error
0.0
0.2
0.4
0.6
Yokoi 2017 * S
Wang 2019 P
Ayaz 2014 P
-6
-4
-2
0
2
4
Standardised Mean Difference

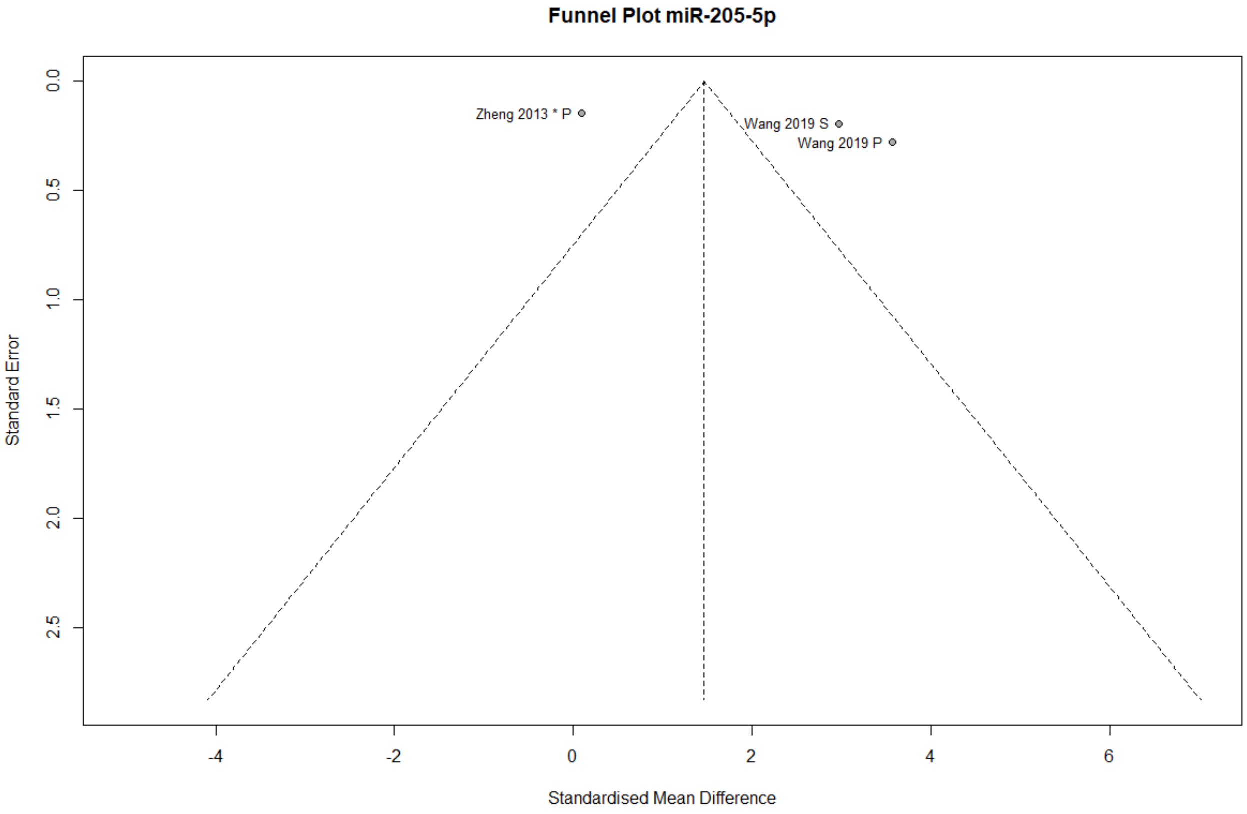
Funnel Plot miR-205-5p
Zheng 2013 * P
Wang 2019 S
Wang 2019 P
Standard Error
0.0
0.5
1.0
1.5
2.0
2.5
-4
-2
0
2
4
6
Standardised Mean Difference

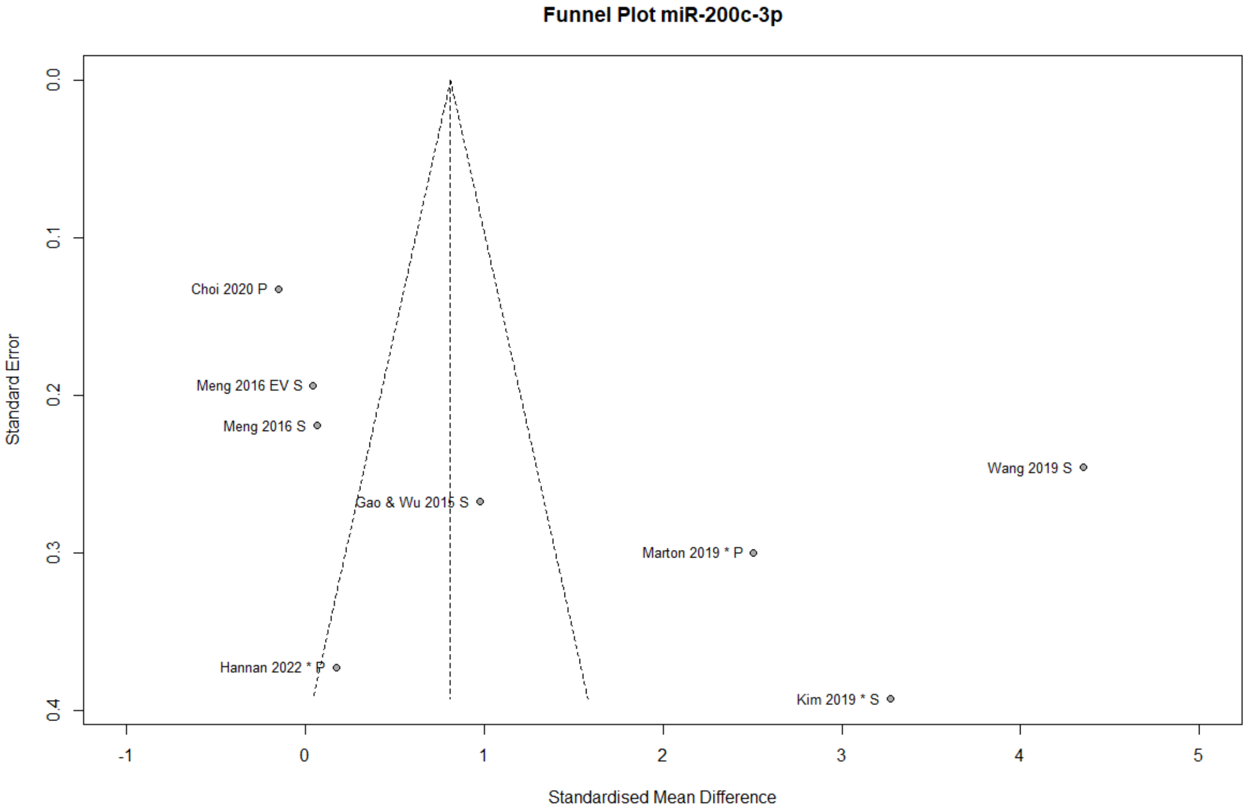
Funnel Plot miR-200c-3p
Choi 2020 P
Meng 2016 EV S
Meng 2016 S
Gao & Wu 2015 S
Hannan 2022 * P
Marton 2019 * P
Wang 2019 S
Kim 2019 * S
Standard Error
0.0
0.1
0.2
0.3
0.4
-1
0
1
2
3
4
5
Standardised Mean Difference

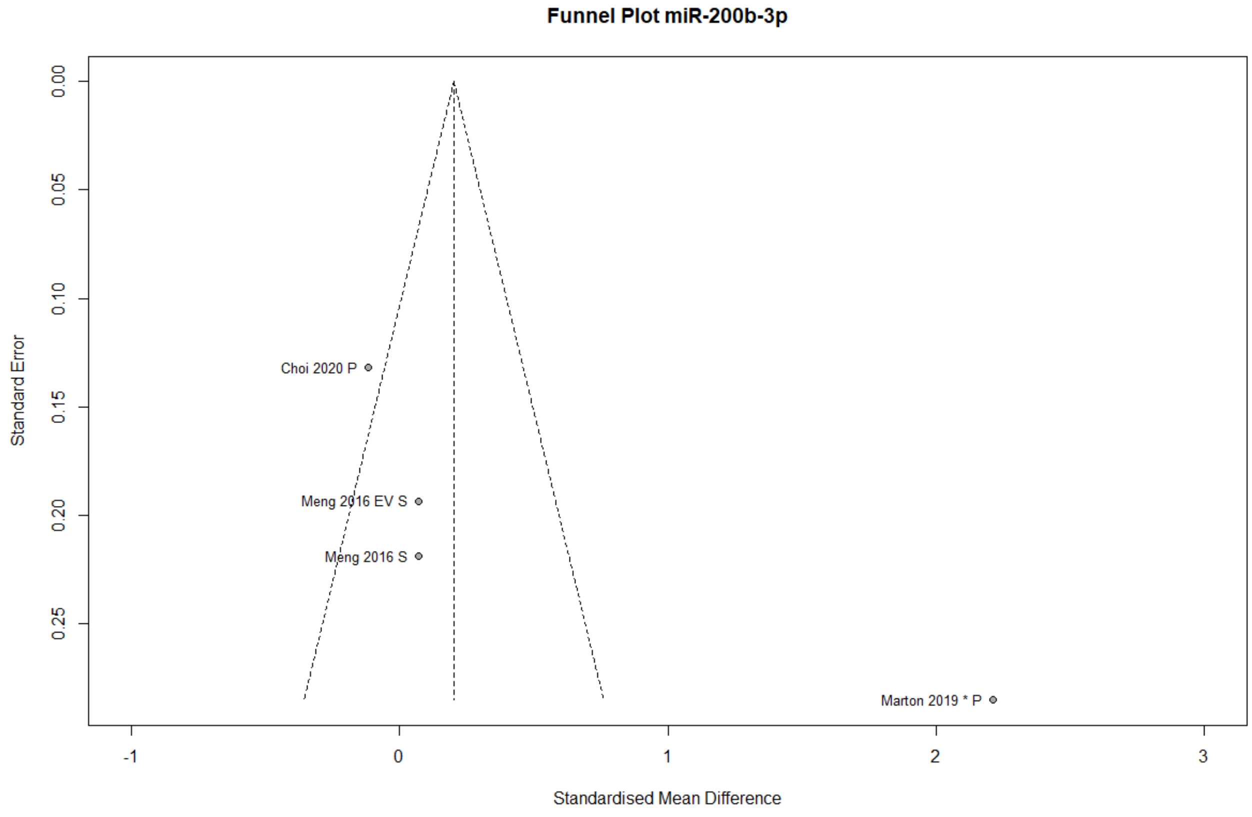
Funnel Plot miR-200b-3p
Standard Error
0.00
0.05
0.10
0.15
0.20
0.25
Choi 2020 P
Meng 2016 EV S
Meng 2016 S
Marton 2019 * P
-1
0
1
2
3
Standardised Mean Difference

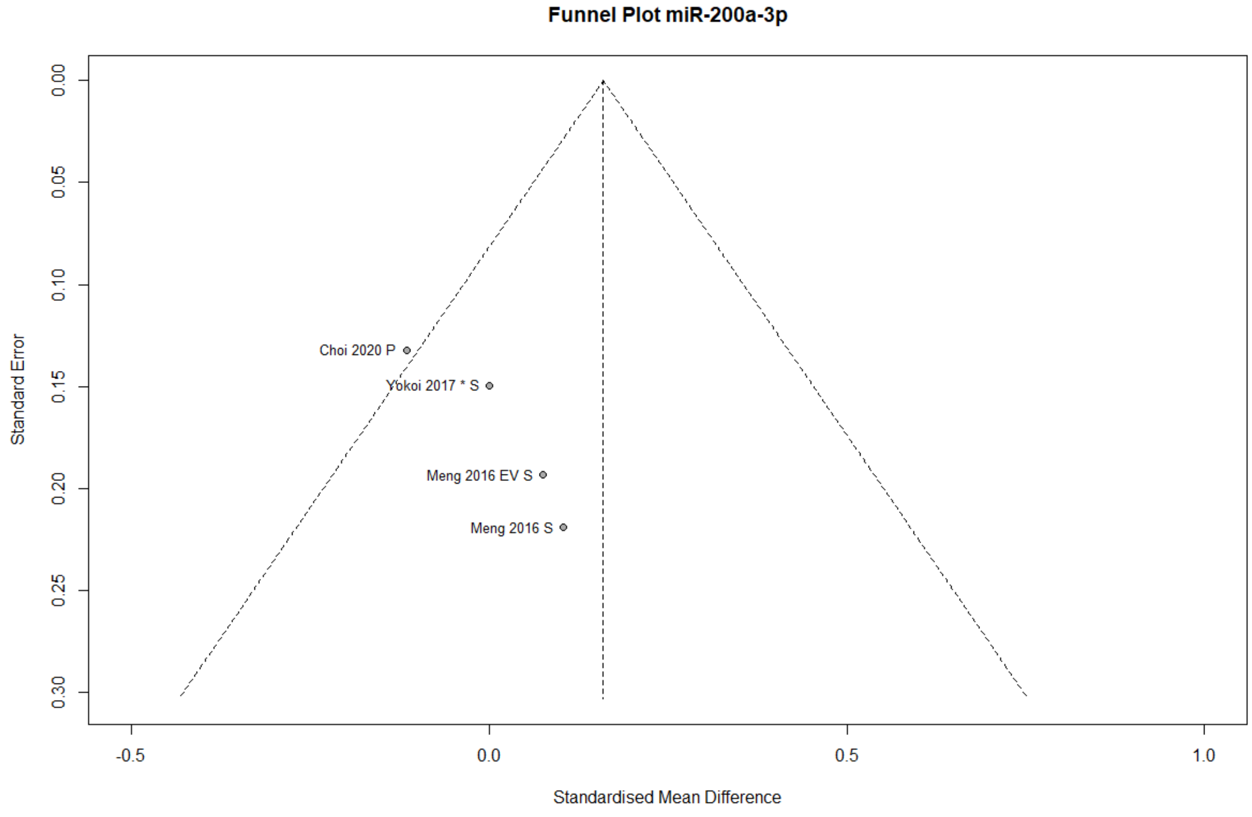
Funnel Plot miR-200a-3p
Standard Error
0.00
0.05
0.10
0.15
0.20
0.25
0.30
Choi 2020 P
Yokoi 2017 * S
Meng 2016 EV S
Meng 2016 S
-0.5
0.0
0.5
1.0
Standardised Mean Difference

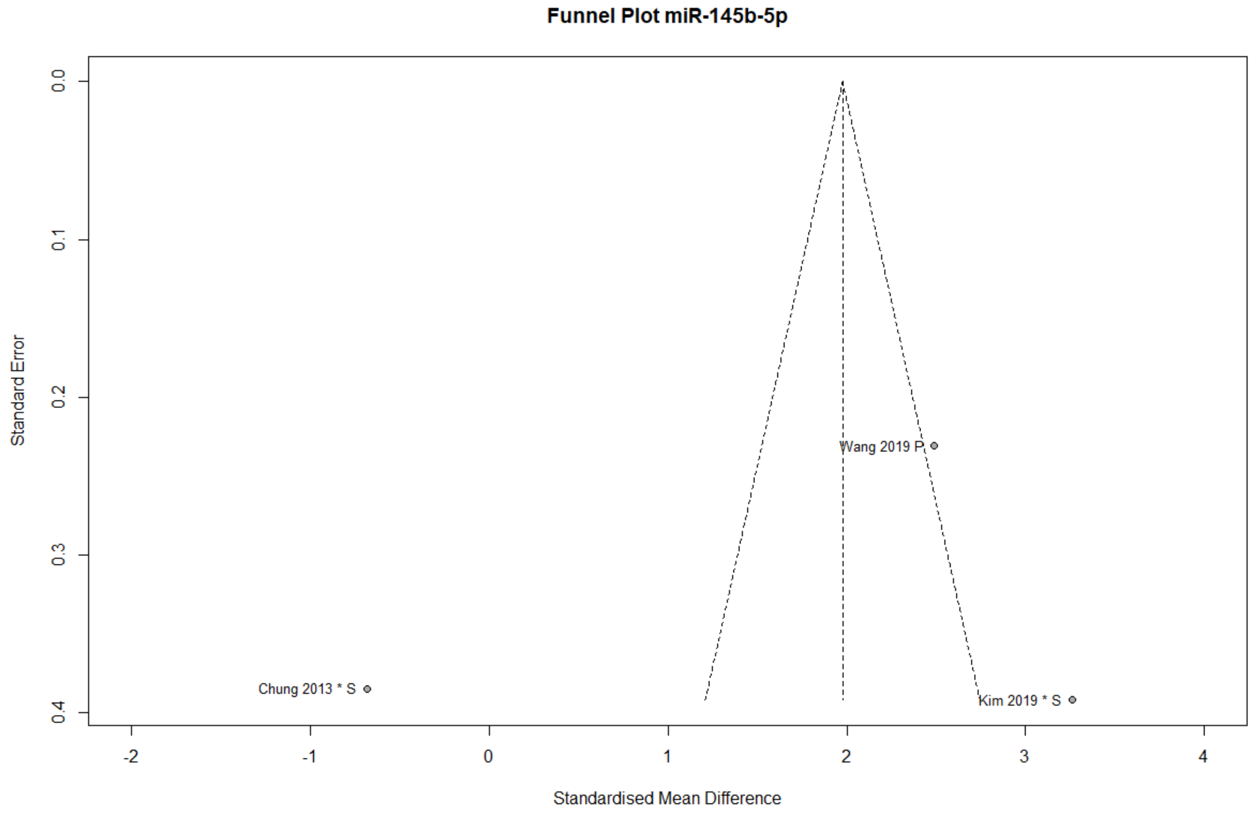
Funnel Plot miR-145b-5p
Standard Error
0.0
0.1
0.2
0.3
0.4
Standardised Mean Difference
-2
-1
0
1
2
3
4
Wang 2019 P
Chung 2013 * S
Kim 2019 * S

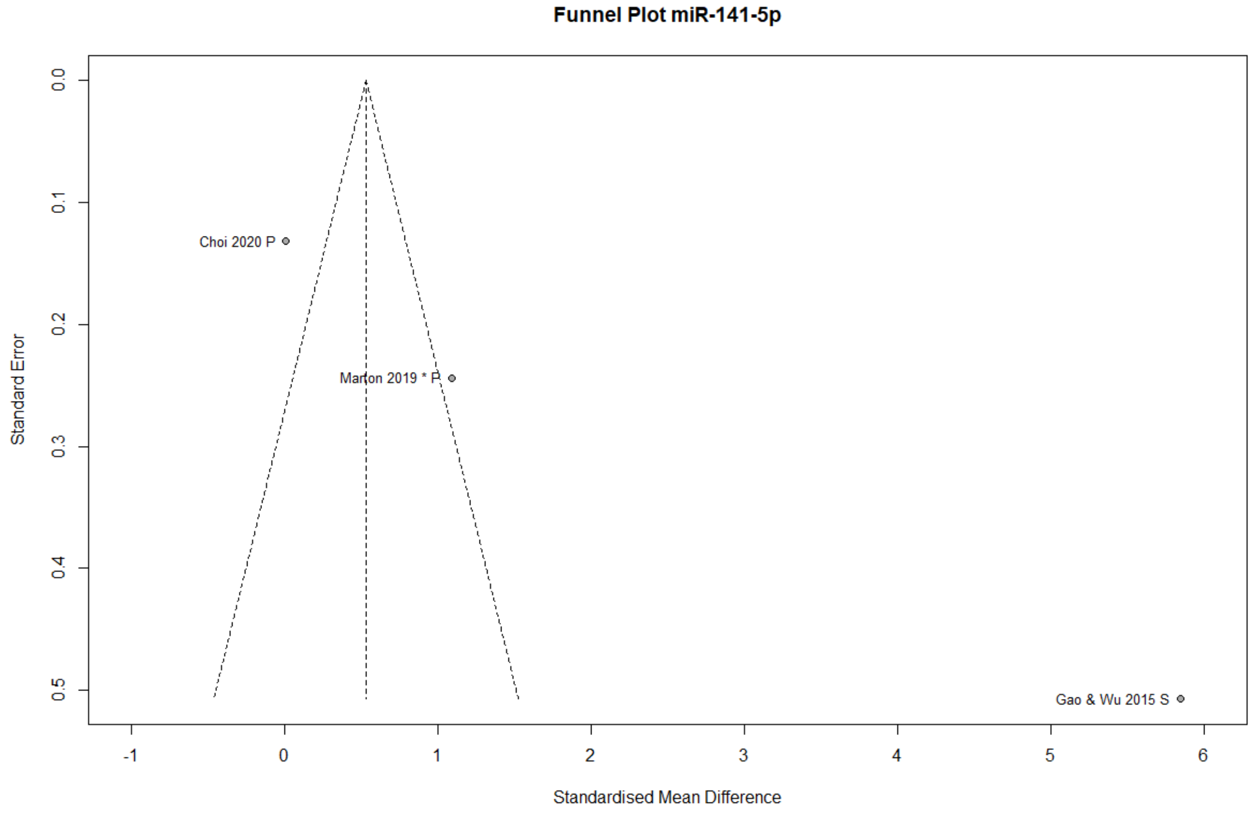
Funnel Plot miR-141-5p
Standard Error
0.0
0.1
0.2
0.3
0.4
0.5
Standardised Mean Difference
-1
0
1
2
3
4
5
6
Choi 2020 P
Marton 2019 * P
Gao & Wu 2015 S

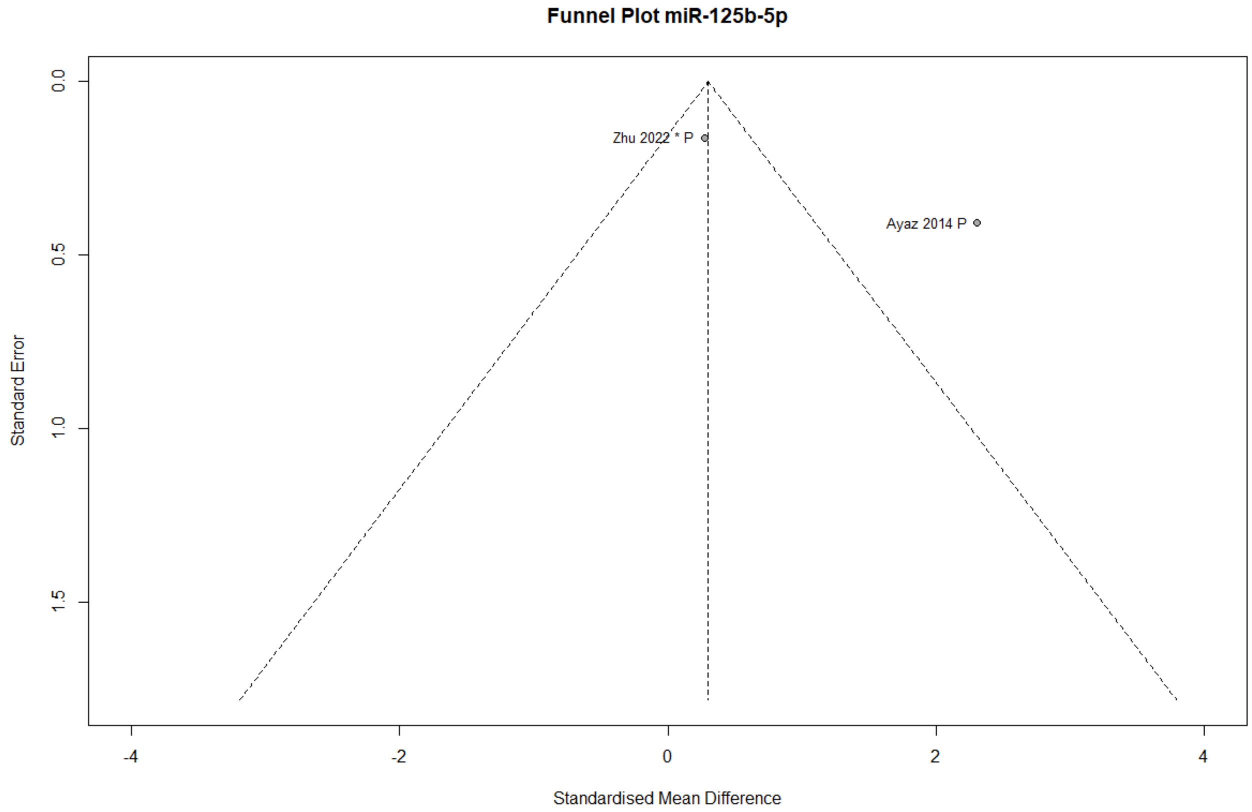
Funnel Plot miR-125b-5p
Zhu 2022 * P
Ayaz 2014 P
Standard Error
0.0
0.5
1.0
1.5
-4
-2
0
2
4
Standardised Mean Difference

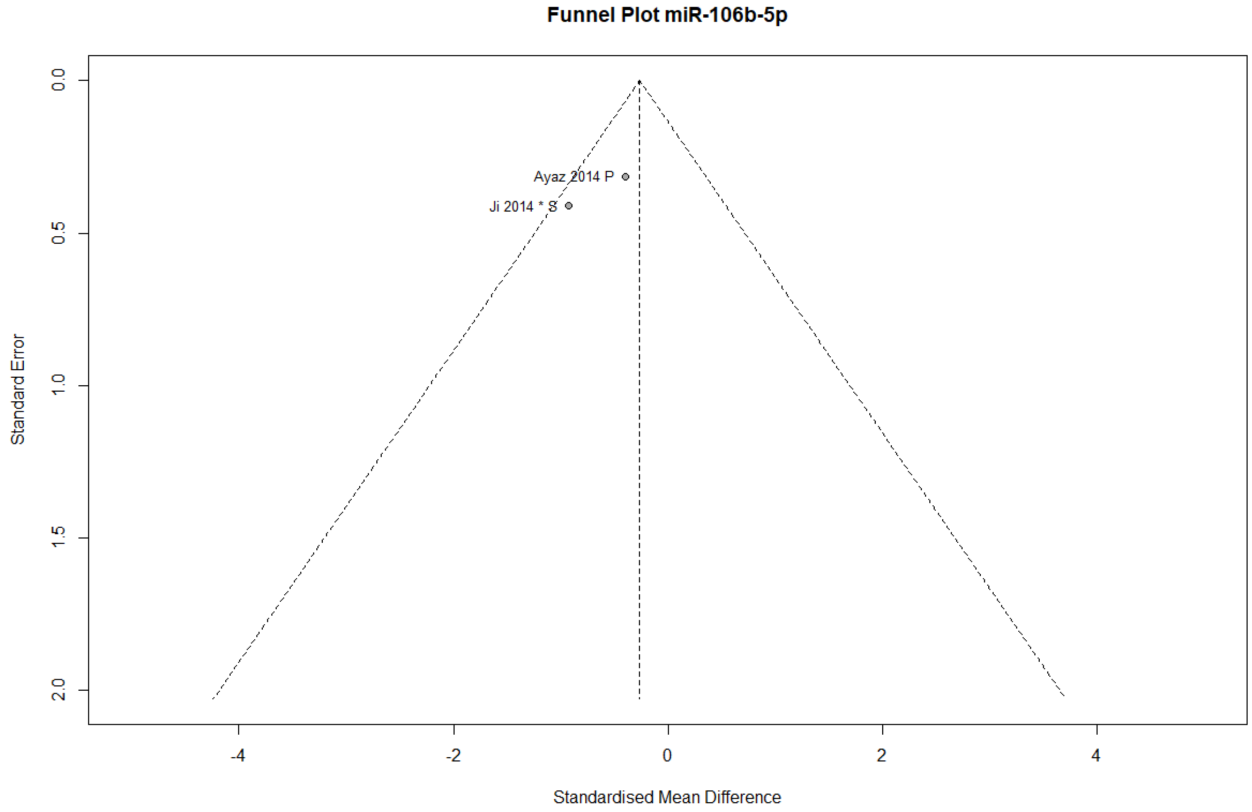
Funnel Plot miR-106b-5p
Ayaz 2014 P
Ji 2014 * S
Standard Error
0.0
0.5
1.0
1.5
2.0
-4
-2
0
2
4
Standardised Mean Difference

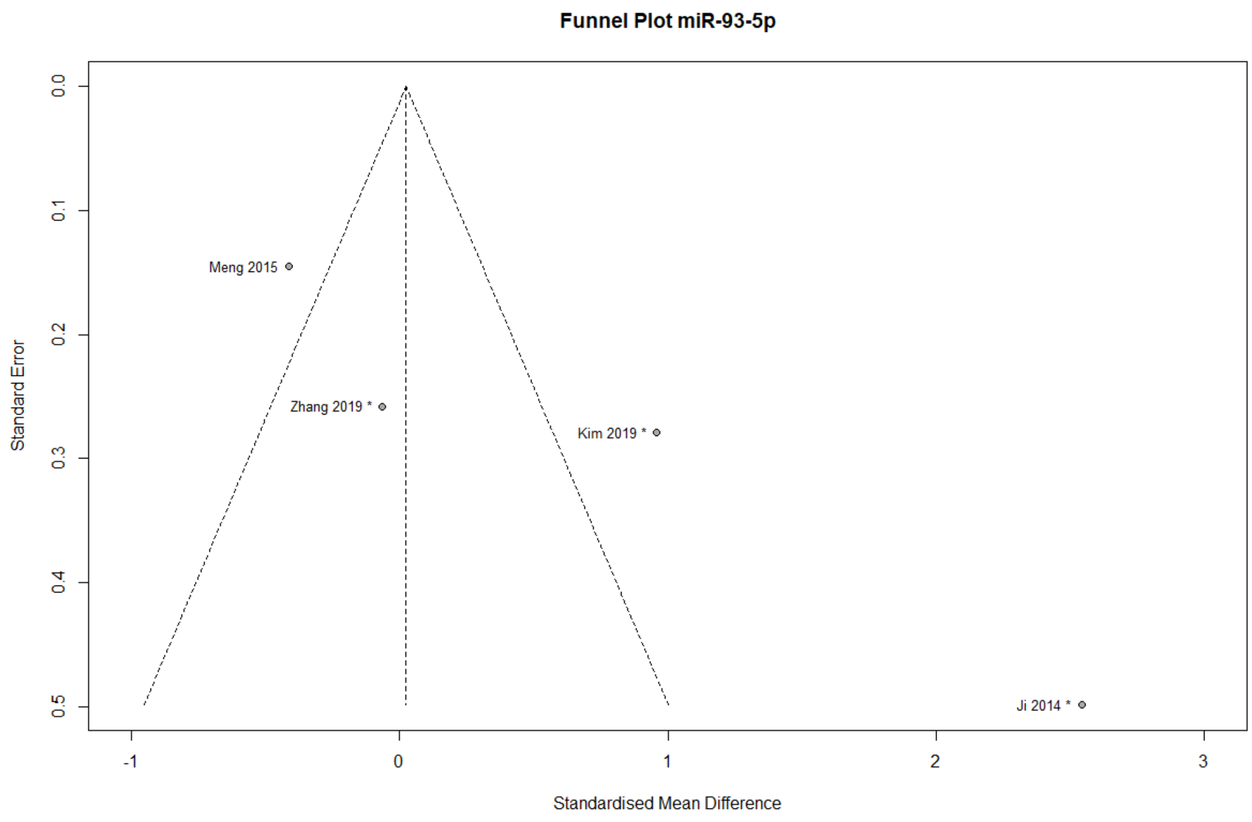

Funnel Plot miR-93-5p
Standard Error
0.0
0.1
0.2
0.3
0.4
0.5
Meng 2015
Zhang 2019 *
Kim 2019 *
Ji 2014 *
-1
0
1
2
3
Standardised Mean Difference

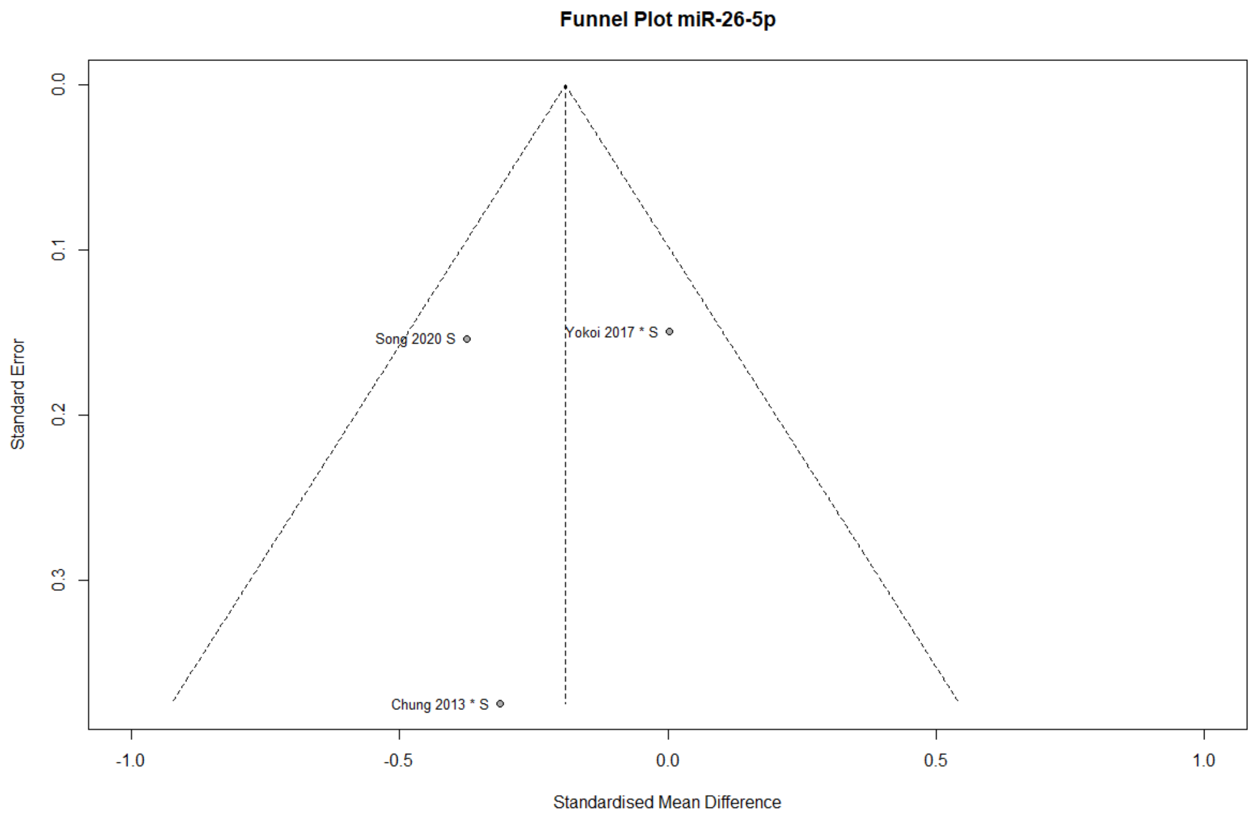

Funnel Plot miR-26-5p
Standard Error
0.0
0.1
0.2
0.3
Song 2020 S
Yokoi 2017 * S
Chung 2013 * S
-1.0
-0.5
0.0
0.5
1.0
Standardised Mean Difference

Funnel Plot miR-21-5p

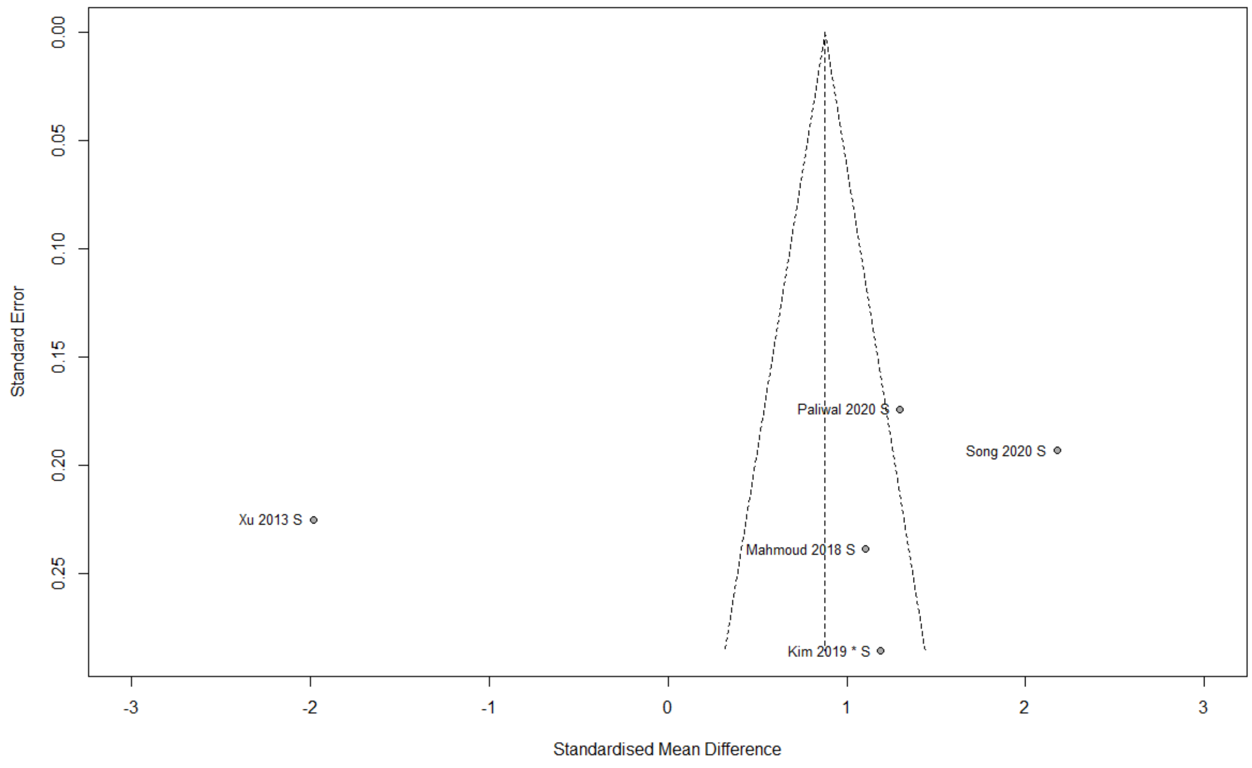

Supplement: Supplementary file 1 [file biomolecules-13-00871-s001.zip › Supplementary Figure S1.pdf]
